# A Novel Method of Treating the Vomiting of Pregnancy

**Published:** 1876-01

**Authors:** 


A Novel Method of Treating the Vomiting of Pregnancy.—Dr. Edward Copeman, President of the British Medical Association, in an article in the *British Medical Journal* of May 15, 1875, relates the histories of three cases in which vomiting had resisted all the usual remedies, and in which a new treatment, discovered by accident, as it were, succeeded in checking the vomiting almost immediately. In the first case, that of a lady six months advanced in pregnancy, the vomiting had become so excessive as to occasion great fears for her safety. Dr. Copeman saw her in consultation with two other practitioners, and advised bringing on premature labor, which the others at first were rather unwilling to agree to on account of her depressed condition, though they finally acquiesced in the plan advised. Accordingly he at once dilated the os uteri as much as he could with the finger, so that he could feel the membranes and head of the child. An attempt was made to rupture the membranes, but failed owing to their flaccid condition and the slight resistance offered by the head to an ordinary female telescopic catheter, the only instrument at hand. After this failure it was decided to wait a little while before resorting to other means. In an hour she was seen again, and he was surprised to learn that a longer period than before had elapsed without sickness, so it was determined to wait another hour in the hope of giving her some nourishment. During that time no vomiting occurred, and it was decided to resort to no further active measures, but to wait for futher developments. No recurrence of the vomiting took place during the night, and the case went on favorably to full term, when she was delivered of a healthy child, and made a good recovery.

The second case was one in which pregnancy was only of two months' standing, and in which the surgeon in attendance had exhausted the best acknowledged remedies, and had arrived at the conclusion that artificial delivery would be necessary to save her life. Dr. C., keeping the first case in his mind, and wondering whether the dilatation of the os in this first case, by removing any undue tension productive of sympathetic irritation of the stomach, had been the cause of relieving the vomiting, examined the uterus, found some degree of anteversion and the os patent enough to admit the tip of the finger. He immediately dilated the os as much as he could, passing his finger all round, and removing all puckering of the os and rendering its edge smooth. She vomited slightly only once after this procedure, and he left her with the understanding that in case the sickness returned he should be summoned again to bring on abortion. But the summons never came, and in a forenight he heard that she began to improve decidedly an hour or two after he left, and that the sickness had entirely ceased. Several times since he has heard that she was doing remarkably well, and he believed that she expected to be confined during the month (May).

In the third case, the patient was the mother of nine children. Generally, during early pregnancy, and sometimes for several months together, she had been troubled with vomiting, but in this pregnancy, for three weeks before his visit, the sickness had been almost constant. She could retain nothing on her stomach, and was in a very weak and enfeebled condition. Considerable albumen, some pus, and a few casts were found in the urine. There was no dropsy. On examining the os, he found it patent, puckered, and dilatable, so he proceeded to dilate it as much as possible with the finger, in the hope that the sickness might be relieved as in the other cases. A few days after this he was informed that no return of sickness had happened since his visit, and that she was able to take food without inconvenience, though she was still very weak and ill. Since then he has learned that she had been safely delivered and was doing well.

In conclusion, he says that the subject seems to him to be of so much importance that he reports these cases without waiting for others, or attempting the *modus operandi* of the treatment, but hopes to communicate further when he has more throughly thought over the subject, and promises to report any future success or failure that may come under his observation.

Dr. Graily Hewitt, in the same journal for May 29, 1875, gives what he considers to be the true solution of the *modus operandi* of treatment in Dr. Copeman's cases. He says that in 1871 he read a paper (see Transactions of the Obstetrical Society, vol. xiii.) in which he enunciated the theory, supported by facts and observations, that vomiting in pregnancy was due to flexion of the uterus, the compression of the tissues at the seat of flexion being the irritation giving rise to the vomiting.

He believes that in all of Dr. Copeman's cases there was acute flexion, and that the dilatation of the cervix relieved the vomiting by overcoming the cramped and confined condition of the uterus; and he believes that this same condition is the cause of vomiting even up to the eighth month, because in such cases the tissues of the uterus at the point of flexion are sometimes left in the early months in a diseased state, being stiffened and unduly resistant, and thus the irritation is kept up. He says that he has been in the habit of treating obstinate vomiting in pregnancy by elevating the body of the uterus, and has found that the same good results have followed as in Dr. Copeman's method.—*British Medical Journal*, May 15 and 20, 1875.—*Canada Medical Record.*

